# Stumblers and Tumblers: Two Pathways to “Unintentional” Fall-Related Traumatic Brain Injury

**DOI:** 10.1089/neur.2020.0033

**Published:** 2021-02-01

**Authors:** Michael D. Cusimano, Melissa B. Korman, George Kazolis, Stanley Zhang, Lorne Tepperman

**Affiliations:** ^1^ Division of Neurosurgery, Department of Surgery, St. Michael's Hospital, University of Toronto, Toronto, Ontario, Canada.; ^2^ Dalla Lana School of Public Health, University of Toronto, Toronto, Ontario, Canada.; ^3^ Keenan Research Centre, St. Michael's Hospital, Toronto, Ontario, Canada.; ^4^ Department of Evaluative Clinical Sciences, Sunnybrook Health Sciences Centre, Toronto, Ontario, Canada.; ^5^ Department of Sociology, University of Toronto, Toronto, Ontario, Canada.

**Keywords:** concussion, early adverse events, fall-related injury, family conflict, life course, traumatic brain injury

## Abstract

Traumatic brain injury (TBI), including concussion, is the commonest neurological condition in high-income countries and is the second commonest condition next to migraines. Although most of these injuries are unintentional, substance abuse and age-related physiological factors have been implicated as causal factors of fall-related TBIs. Our study used qualitative methods and a life course perspective to examine whether life events and psychosocial antecedents, such as early adverse childhood experiences, play a role in the occurrence of non-intentional fall-related TBI. In-depth interviews were conducted with 27 patients who sustained a TBI due to unintentional falls. Transcripts were qualitatively analyzed to explore factors related to their prior life experiences that may have been related to the reasons that led to their falls. The results reveal that childhood family conflict and peer-influenced risky behaviors may have contributed to poorer mental and physical health in adulthood, which in turn contributed to injuries. Respondents whose behaviors did not play a direct role in their injury event were labeled “Stumblers.” These patients' falls were seen as being related to unfortunate unique environmental and situational factors and could colloquially be described as “accidental falls.” We also identified a distinct group of patients who had a cumulative life experience starting in early childhood that contributed to a pattern of riskier behaviors, ultimately culminating in a fall-related TBI. The second group of patients were labeled “Tumblers” as they chose to participate in risky activities, regardless of whether they considered them to be risky, which ultimately led to the fall-related TBI. This group was identified by a purposeful volitional state that sought out the “opportunity for accidental fall.” Childhood family conflict and peer-influenced risky behaviors were important precursors to mental and physical health states in this group.

## Introduction

Traumatic brain injury (TBI), including concussion, is a serious global public health problem. The incidence of TBI has been increasing worldwide,^1–3^ with an estimated 10 million cases annually.^3^ In Canada, TBI is the second most prevalent neurological condition.^4^ Many of those who sustain TBI experience lifelong physical, cognitive, behavioral, and/or psychosocial impairments as well as economic burdens associated with their injuries.^5,6^

Causes of TBI can be classified as unintentional and intentional, with the commonest cause, falls,^7^ being traditionally considered unintentional. Age is a well-known risk factor for fall-related TBIs. Indeed, our previous research reported that all age groups 65 years and older experienced significant increases in fall-related TBI rate over an extended study period (2002–2017).^8^ Alcohol use,^9^ substance use disorders,^10,11^ and violent behaviors are frequently associated with intentional TBI (e.g., assaults). In this study, we asked whether events experienced over the life course of the person sustaining a TBI could have influenced the occurrence of an apparent unintentional TBI related to a fall.

The victim precipitation theory helps us understand both intentional and unintentional TBI. It suggests that people cause or initiate confrontations that may eventually lead to their own injury or death.^12^ Precipitation can be passive or active. During *passive* precipitation, the victim unknowingly instigates or encourages the event. Conversely, during *active* precipitation the victim knowingly provokes the event.^11^ There are three types of victims: *Latent*, *Provocative*, and *Participating.*^13^ Latent victims are more likely than others to be victims due to predispositions or character traits. Provocative victims play an important part in the origin of the event. Participating victims play their part while the event is occurring by being inattentive, or even by assisting in the process. These categorizations can be further divided into Dormant and Active victims. Dormant victims play a passive role in their victimization. Therefore, Latent victims fall into this category. Conversely, Active victims include Provocative and Participating victims because they are instrumental in facilitating the event. Victim precipitation theory contributes to our understanding of pathways through which people become victims of injury. However, we do not fully know *why* some people exhibit certain behaviors, such as engaging in risky activities.

To better understand the etiology of fall-based TBI and develop appropriate rehabilitation and prevention strategies, we explored how the risk factors for injury may have emerged. According to a model of sport injury,^14^ both intrinsic and extrinsic risk factors interact and accumulate, rendering the individual “an accident waiting for a place to happen”; also known as a “nearly sufficient constellation” of causal factors.^15^ The final straw for causation is the inciting event, which is clearly or visibly related to the injury, and is usually regarded as the cause.^16^ This event is not the only component of injury causation and may not be the most important factor.^14^ In this work, we expanded these ideas to discover and analyze the intrinsic and extrinsic factors that interact over the life course of an individual to create the “susceptible faller” by understanding the types of life events that create vulnerability to TBI using a life course perspective.

Through sequences of experiences that extend across an individual's life,^6^ known as trajectories, the life course perspective suggests that occurrences in childhood, external and internal factors, can influence individual development and shape patterns for future experiences.^17,18^ This perspective also considers social aspects of an individual's life, suggesting that people in important relationships (e.g., family and friends) occupy mutually influential and interlocking developmental trajectories.^6,19^ The life course perspective also reveals heterogeneity between the lives of individuals who experience childhood family conflict and adverse early events such as abuse and neglect.^20^

A substantial amount of work has examined how age-based factors, physiological risk factors, and drug and alcohol abuse are associated with accidental falls and TBI. Research indicates that age affects both risk of falling and the severity of injury, as older people are more likely to suffer from complications of injury.^21^ Further, research illustrates that 44–79% of people with TBI have a history of alcohol abuse,^22,23^ and 21–37% report a history of illicit drug use.^22,24,25^ Studies have also revealed evidence of a complex relationship between substance abuse, family dynamics, and TBI,^26–28^ which will be explored in this research. Minimal attention has been devoted to identifying the socio-developmental factors that alter the life course of individuals and potentially contribute to their eventual injury. By determining the early factors that may set a child on a trajectory that leads them to become a victim of TBI or other injuries, the current study strives to provide important knowledge for understanding the development of health behaviors, the effects of early adverse events, and the appropriate rehabilitation of patients with fall-related TBI. Further, information obtained in this study can help develop policies related to early child health that could potentially pay dividends later in the prevention of adverse events such as TBI.

## Methods

### Ethics approval

The Research Ethics Board of St. Michael's Hospital provided approval for this study.

### Recruitment and data collection

A total of 27 participants were recruited from St. Michael's Hospital, a major regional trauma center in downtown Toronto, Ontario. Participants were included in this study if they were admitted to the hospital with a Glasgow Coma Scale score of 13 or lower after a fall that occurred within the prior 1–3 years but at least 3 months after the fall occurred. In other words, participants had to achieve a phase of stability in their medical conditions after the fall, and the minimum time after sustaining the fall to the time of participation in the study was 3 months. All participants had achieved stability medically prior to participation, and all participants started the study during the interval between 1 and 3 years after their fall. Face-to-face structured, open-ended interviews that explored developmental trajectory including relationships with family and peers, education, career, prior TBI, mental health status, criminal or delinquent events, prior violent episodes, and the use of alcohol and drugs were conducted. All interviews were tape-recorded and transcribed.

### Statistical analysis

**FIG. 1.**
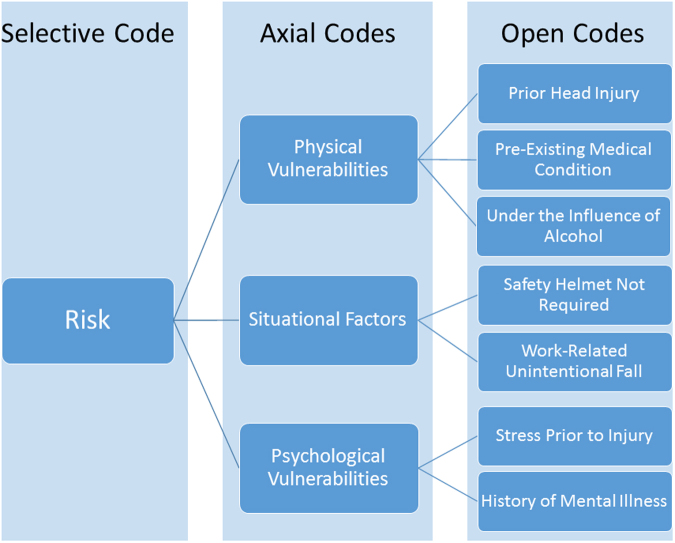
Partial coding flowchart for pathway titled Stumblers Pathway, which included codes related to those at risk for fall-related TBI. TBI, traumatic brain injury.

**FIG. 2.**
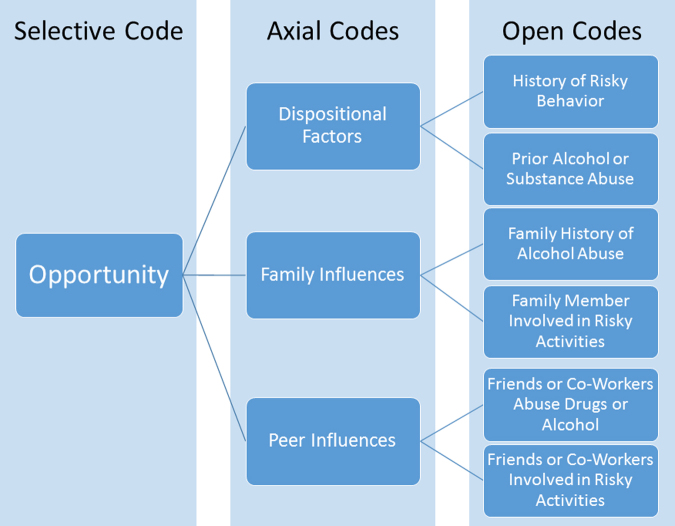
Partial coding flowchart for pathway titled Tumblers Pathway, which included codes related to opportunity for fall-related TBI. TBI, traumatic brain injury.

NVivo 10 software, specifically the code families and network generation features, was used for interview transcript analysis. The constant comparative method was employed,^22,27–30^ whereby line, sentence, and paragraph segments of the transcribed interviews were reviewed and assigned fitting codes. Each transcript was independently analyzed by two members of the research team. The coding process involved the generation of codes that led to categories, and finally themes that helped formulate a theory. Three coding steps were used in this process that consisted of *open coding*, *axial coding*, and *selective coding* (Figs. 1 and 2). Any differences in coding were discussed, and the original field notes were reviewed to reach agreement on the most appropriate classification. The codes and categories were adjusted throughout the coding process as themes emerged.

Trustworthiness was addressed using two main techniques: 1) the specific grounded theory method incorporated into this study helped achieve the *Credibility* measure by providing “rich” descriptions of the findings, and (2) the rigorous methodological structure and execution of the coding process in NVivo helped with *Transferability*.

## Results

Of the 27 total participants, 18 were male (mean age 42.5 years, standard deviation [SD] = 13.60) and 9 were female (mean age 46.7 years, SD = 15.04).

The multi-stage coding process led to the generation of codes, categories, and themes. Sixty-one different codes were generated and then collapsed into more encompassing categories which were further broken down into two dominant themes: “risk” of accidental falls labeled as the “Stumblers” group and “opportunity” for accidental falls, labeled as “Tumblers.” Flowcharts illustrating how the two overarching themes emerged are provided in Table 1 and Figures 1 and 2. The *Risk Theme* includes three categories: physical vulnerabilities, situational factors, and psychological vulnerabilities. The *Opportunity Theme* comprises three other categories: dispositional factors, family influences, and peer influences.

**Table 1.** Level 1 Codes (Open Coding)

Code	# of sources	# of references
Prior history of risky behavior	23	119
Prior alcohol or substance abuse	17	52
Participant's friends or co-workers abuse recreational drugs or alcohol	16	27
Participant believes they are suffering from memory deficits since the accident	14	18
Participant has displayed more emotional or irritable behavior since the accident	12	25
Participant accepts responsibility for causing their accident	12	23
Family history of alcohol abuse	12	22
Participant's friends or co-workers involved in risky activities	11	21
Participant had suffered a head injury in the past	11	13
Participant believes they are suffering from deficits in attention or concentration since the accident	11	12
Participant believes there is nothing they could have done to prevent the accident	11	12
Participant believes their recent head injury could have been avoided	11	14

Codes ranked in (descending) order of linkages.

### Risk theme: Stumblers

#### Physical vulnerabilities

This category was coded in 19 of 27 transcripts. Almost half of the participants spoke of previous head injuries. One-third of the participants, approximately half of those with physical vulnerabilities, revealed medical conditions that existed prior to the injury event.

“*It's because I am diabetic and I was diabetic before the accident. And I was, I mean—the doctor… said that probably I had a weakness in my blood sugar level … and I just lost my control but I don't remember.”*

An equal number of participants revealed that they were under the influence of alcohol prior to the injury event.

*“We were pre-drinking on the way and then we got there—we continued to drink at the bar. The last thing I remember is buying a drink for a friend of mine and I went to go to use the washroom … I don't remember anything else.”*

#### Situational factors

Situational factors were cited in more than half of the transcripts. More than one-third of participants believed that their injury was due to external circumstances.

*“I definitely wouldn't blame anyone because accidents are accidents. Do you think the person meant to step on my coat? No. It was my stupidity for having a coat that was that long and falling behind the stair behind me.”*

Further, a small number of participants (5 of 27) admitted their injury was related to them not wearing a helmet and four participants stated that their fall was work-related.

*“Well, I work at a place that hard hats are not required and if I had fallen the way they said, the hard hat would've come off before I fell, so…”*

#### Psychological vulnerabilities

Psychological vulnerabilities, such as stress and sleep deprivation, were identified within one-third of the transcripts.

*“Prior to the accident, I was not sleeping well. I seem to fall asleep for 2 or 3 hours … My son had recently moved to Singapore to start his new business and that was probably wearing on me.”*

### Opportunity theme: Tumblers

The life histories of Tumblers reveal key developmental and psychosocial factors related to their injury, including: 1) negative, abusive, or delinquent family and peer relationships that shaped their current lifestyle; 2) early exposure to substance abuse and risky activities; and 3) willingness to imitate these behaviors.

Of 27 respondents sampled, 23 had a history of risky behavior (e.g., delinquency, fighting, and aggressive driving). Of these 23 participants, 16 also had a history of substance abuse and/or peers with a history of substance abuse. Among these 16 participants, 10 indicated a family history of substance abuse and/or peers with a history of risky behavior. Thus, 10 of 27 respondents can be viewed as coming from a family environment of substance abuse that also includes personal substance abuse. Yet only 5 of 10 (50%) of these respondents accepted any responsibility for their accident or believed they could have prevented it.

#### Dispositional factors

A range of dispositional factors appeared to play a role in the falls of all 27 participants. However, prior history of risky behavior (23/27) and prior alcohol or substance abuse (17/27) were most frequently reported.

*“But I do a lot of crazy stuff. I was a courier for 12 years, skydive, rock climb…”*

*“Before the head injury, I was drinking a whole lot—I've been drinking since I was a kid…”*

#### Family Influences

The majority (78%) of participants in this study spoke of their families and the influence they had on their behaviors and emotions. Common topics included alcoholic parents, severe punishment by parents, poor relationships with parents, neglect, abuse, overly controlling parents, parents with limited involvement, and family members engaging in risky or delinquent activities.

*“My brother was a bad boy so there's that… most of his friends I don't hang out with them, because I'll go to jail if I hang out with them.”*

Approximately half of the participants described a history of familial alcohol abuse:

*“Yep. My uncles—they were all alcoholics. My grandfather. And my mother's brothers. Yeah I was surrounded. Growing up it was rampant.”*

A smaller number of participants, about one-quarter, spoke of both childhood psychological (verbal conflict) and physical (including sexual) abuse at home:

*“My father was very abusive and he was beating us—I was about 8 years old—7, 8 years old. And the pain was so excruciating—I will never forget that.”*

#### Peer influences

Peer influences were coded in a large majority of the transcripts. This category was dominated by friends or co-workers with substance abuse issues, and friends or co-workers who were involved in risky activities. Participants stated that they *“normally drink with friends.”* When discussing the problems that alcohol caused for their friends, one participant stated: *“Health-wise yeah, quite a few of them died.”* Among those who had friends or co-workers who were involved in risky activities, one participant stated that most of the injuries were sports-related:

*“Yeah definitely sports injury. Multiple times with ankle, ugh broken legs. A lot of them are—even without revealing their names, broken wrist, again sports injury too. A lot of it has to do with sports injuries.”*

Some transcripts revealed an overlapping of these codes whereby injuries occurred while the participant was under the influence of drugs and/or alcohol and partaking in risky activities with their friends.

*“We took straight shots and we finished it and then after that uh … we ended up doing pretty much what we always do which is like smoke some weed ya know? … So we had no where to go smoke. So … like … one of our ideas were … to probably go smoke inside the bus … there was like three of four buses parked beside each—the first bus didn't have an emergency exit so … we saw that the second bus had one so everyone jumped over … And I believe there was like ice on there because you know how the top of the bus is rounded at the top, so my butt slipped off that so there was ice on there and stuff. I don't even know what truly happened at that point.”*

## Discussion

Our results revealed two main pathways for fall-related TBI. The *Stumbler Pathway* reflects a pathway in which those who fell played a passive role in their TBI event, as their falls were found to be related to physical vulnerabilities, situational influences, and psychological vulnerabilities. In contrast, the *Tumbler Pathway* describes a pathway in which those who fell played an active role in their TBI event through dispositional factors, and family and peer influences. The life trajectories of Tumblers revealed key developmental and psychosocial factors related to their fall-related TBI, including adverse childhood events and early socializations, which we believe created personal vulnerabilities and behaviors that led to hurting oneself. Although members of the Tumbler Pathway group were aware of their risk-taking, many were unaware of the role they played in their own injury.

### The Stumbler pathway

Among the group of Stumblers was a predominance of themes related to age, balance, pre-injury alcohol levels, polypharmacy, and environmental hazards. These factors emerged during analysis as the Risk Theme and included three categories: physical vulnerabilities, situational factors, and psychological vulnerabilities. These latent factors were directly related to the injury event, so we called this group of patients Stumblers. This group reinforced previous findings from established literature that indicate a vulnerability to falls.^7,10,11,21^

#### Physical vulnerabilities

Participants within this category referenced pre-existing medical conditions and/or alcohol consumption prior to their fall-based TBI. This suggests that a subset of participants within this group were physically vulnerable to their accidental fall. The codes within the physical vulnerabilities category align with the emergent Risk Theme found in our analysis, which suggests that a subset of victims presented latent or dormant vulnerabilities to fall-based TBI. This finding reinforces previous literature that discusses certain physical vulnerabilities (e.g., alcohol consumption) and their link with increased rates of TBI.

#### Situational factors

According to our results, recurring situational factors (external variables) played a role in the fall-based TBI for a subset of participants. Situational factors included lack of safety equipment and occupation-related injuries. Based on their extrinsic and involuntary aspects, situational factors fall within the Risk Theme, which affects the passive Stumblers.

#### Psychological vulnerabilities

A number of participants referenced psychological vulnerabilities (which are intrinsic risk factors) prior to their fall-based TBI. This suggests a passive susceptibility to injury, similar to the other two categories within this theme.

**FIG. 3.**
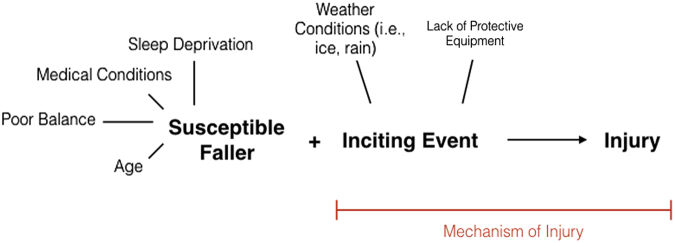
A model for unintentional fall-related TBI characteristic of the Stumblers Pathway. TBI, traumatic brain injury.

The Risk Theme of the Stumbler Pathway confirms many causal factors previously identified in TBI research; but the discovery that multiple factors from this theme affected the participants demonstrates that these risk factors may affect, and occur with, each other. We used our findings to develop a new model (Fig. 3) to explore causation in fall-related TBI. This model assumes that each variable is not isolated and demonstrates how variables likely work together to create the susceptible faller.

### The Tumbler pathway

Tumblers, which we defined as individuals who played an active role in their fall-related TBI event through their involvement in risky and/or delinquent behaviors with origins dating back to adverse early developmental childhood and early family experiences, demonstrated combinations of Provocative and Participating victim typologies. Although these participants played an active role in their TBI, our results showed that Tumblers had little insight into their role in their own injury. Our research identified subtexts in participant's psychosocial histories that contributed to their injury through associations with risky behaviors, activities, and lifestyles. These patterns were captured by the dispositional factors and family/peer influences categories that emerged from our qualitative analysis as the Opportunity Theme. Although dispositional factors have been recognized in previous TBI literature,^22–25^ family and peer influences are not well-documented in relation to TBI. Our findings, as indicated by respondents' early exposure to substance/alcohol abuse, dysfunctional early life environments, and risky activities suggest that negative, abusive, or delinquent family and/or peer relationships were instrumental in shaping the lifestyles, development, and decisions of Tumblers.

#### Dispositional factors

This category falls within the Opportunity Theme for accidental falls theme due to its focus on behavior and activity patterns. Our findings were consistent with previous findings that most TBI victims have a history of substance abuse.^22,23^ Although prevalent in previous literature, this code was not as prevalent in the transcripts as a prior history of risky behavior. This new finding reveals that risk-takers may be more susceptible to TBI than those with substance abuse issues. The combination of these two factors seems very likely to create a situation highly likely to be related to TBI, as both codes were found in more than half of the transcripts.

#### Family influences

The prominence of the family influences category within the transcripts suggests that family environment may be a precursor to later involvement in risky activities. Reflecting on the life course perspective, it was found that early family relationships played an intermediary role in changing the life trajectories of those who sustained falls, and in modeling future relationships and lifestyle choices. This may have indirectly contributed to later injury events experienced by Tumblers through associations with substance abuse and/or delinquent peer relationships. Research suggests that repeated exposure to unpredictable stress, such as chronic childhood family conflict, can damage areas of the central nervous system, and may be associated with poorer mental and physical health in adulthood through a variety of mechanisms.^31–36^

The life course perspective presents a new, indirect route through which childhood family conflict might threaten adult health outcomes. A large portion of youth who present aggressive behaviors continue on a path of violence into adulthood.^37,38^ This suggests a “life course persistent development pathway.”^38^ The analysis of transcript histories revealed important family and peer influences that may have altered the individuals' life course trajectories, subsequently influencing the risk of TBI. Research has consistently shown that family environment is a crucial factor in determining and predicting adolescent delinquency, and a lack of family cohesion has been found to be significantly related to the development of delinquent behaviors.^39^ The interaction of family and peer influences emerged as a central component of the Opportunity Theme.

#### Peer influences

A number of participants in this study acknowledged the impact peers had on their engagement in risky behaviors, which in turn, contributed to their fall-related TBI. Positive family relations can aid in reducing the effects of an adolescent's associations with peers who are committing delinquent acts.^40^

Through the concept of trajectories, we found that childhood events shaped patterns of individuals' experiences *beyond* childhood and across adulthood. Experiences of family abuse and harmful peer relationships occurring earlier in the life course affected later outcomes, such as making risky choices, which contributed to a TBI event later in life, through a process best described as cumulative disadvantage.^5^

Early experiences formed a set of characteristics that are common to the Tumbler profile including: history of risky behavior and/or peers with a history of risky behaviors, history of substance abuse and/or peers with a history of substance abuse, and family history of substance abuse. Of the 27 participants, 10 revealed all these characteristics in their interviews; however, only half of these participants accepted any responsibility for their accident or believed they could have prevented it.

**FIG. 4.**
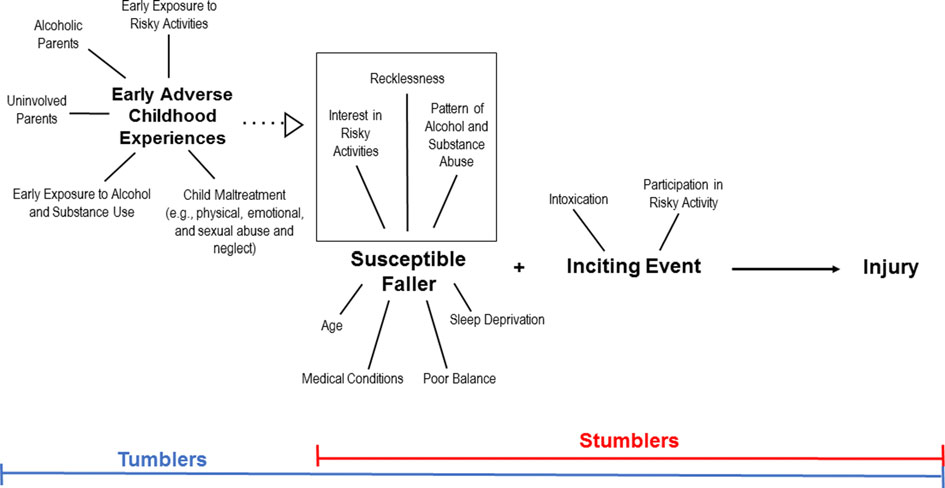
Model of pathway that leads to injuries for both the Stumblers and Tumblers Pathways. The model does not necessarily have to be linear.

Assimilating these qualitative findings into our fall-based TBI model (Fig. 3), and following a life course perspective, an integrative mechanism through which Tumblers suffered their injury event is proposed in Figure 4. The significant psychosocial influence of early family and peer relationships, and the consequential change in trajectories that led to increased exposure to substance abuse (intrinsic factor) and/or risky activities (extrinsic factor), may have played a fundamental role in the fall-based TBI event of Tumblers.

### Undercurrents of violence

A subtle pattern that emerged throughout the transcripts of Tumblers was their exposure to physical, psychological, emotional, or sexual violence. This may aid in understanding why these participants engaged in risky behaviors. Family violence and child maltreatment are at epidemic proportions worldwide.^41^ Research clearly shows that children are negatively impacted by physical and verbal abuse directed at them, and exposure to violence within the family.^42^

Limited family connection and negative peer influence were also identified as important themes by participants within the Tumblers group, and emerged as contributing factors to their involvement in risky behaviors. The literature supports the importance of these connections, identifying parental support, authoritative parenting, and healthy family cohesion as protective factors.^43^ Parenting style, along with the presence or absence of parental figures in their lives, was discussed by several participants in this study. Research shows that a lack of positive adult engagement can lead adolescents to befriend other rejected and deviant peers, and these relationships can lead to violence and criminal behaviors.^44^ These violent behaviors or lifestyles may have served as precipitating factors in the TBI victims' injury event (Fig. 4). The lack of protective factors can influence life course trajectories, which may in turn influence the likelihood of sustaining a TBI.

Children who have been exposed to and/or victimized by family violence exhibit significant levels of internalizing and externalizing behaviors.^45–47^ A subset of participants within the Tumblers group indicated verbal conflict and abuse within their family histories. Findings of both clinical and empirical research suggest that children exposed to violence within the home will experience “adaptational deficits.”^48^ As such, our group of Tumblers may have been more susceptible to their TBI as a consequence of such deficits and behavior patterns that lead to risky activities. Children who have experienced violence may become completely desensitized to the effects of violence and eventually assume the role of the aggressor.^49^ This desensitization toward risky activities may have facilitated Tumblers' active role in entering risky social and behavioral contexts that ultimately contributed to injury. Further, because TBI events may contribute to physical, cognitive, behavioral, and/or psychosocial impairments,^5,6^ these events may allow an already at-risk Tumbler to experience further risky health events, such as future TBI or violence.

### Limitations

Our study has several limitations. First, our study was based on a small sample of patients with TBI. Due to the small sample size and the use of purposive sampling of only patients with TBI, the generalizability of our findings is limited. Second, although unintentional injury may be particularly common in risky locations, often there are no victimizers or perpetrators. Indeed, the participant may be both the victim and perpetrator by putting themselves into the risky situation. Finally, although we identified two distinct pathways, victims of falls may demonstrate features of both, a concept that was not explored in this study. In spite of these limitations, the results of these analyses represent an important contribution to the literature, and promote future research into the biopsychosocial aspect of TBI and the creation of preventative measures for TBI. To our knowledge, this is the first study to use a life course perspective to examine whether life events and psychosocial antecedents, such as early adverse childhood experiences, play a role in the occurrence of unintentional fall-related TBI. As such, further investigation of these concepts in future studies is needed to confirm the validity of the results.

## Conclusion

Two pathways to fall-related TBI were identified: 1) the traditional Stumbler Pathway associated with physical and psychological vulnerabilities and situational factors, and 2) a novel Tumbler Pathway reflecting longer-term family and peer influences and dispositional factors related to active risk-taking. Past adverse early childhood experiences such as family abuse and peer delinquency in the lives of Tumblers constituted risk factors and set into motion sequences of experiences and activities that extended and accumulated over time. This, according to life course theory, created a cumulative disadvantage that led to actively making riskier choices with little insight into potentially adverse consequences, which may explain these individuals' TBI later in life. To this end, Tumblers (active fallers) played an important role in the precipitation of their own injury. This is not to imply blame, but rather to acknowledge that their life course trajectories were negatively altered by early family and peer influences, leading to increased involvement in substance abuse and risky activities, which ultimately may have contributed to their injury event.

This finding is important for several reasons. First, it strengthens our understanding of how victims may precipitate their own injury, however unwittingly, and provides support for the victim precipitation theory. Second, it reveals how we can use a life course perspective to link many of the already established injury risk factors together. The life course perspective provides a framework that allows for the integration of multiple disciplines, and draws attention to the multi-faceted pathogenesis of injuries. This approach opens up new avenues for injury research, extending the linear, univariate analyses relied upon in past TBI studies. In addition to the potential effects on the health and safety of future generations, this work contributes to the scientific literature by revealing correlations between TBI and psychosocial factors, which should be studied further.

Effective rehabilitation requires a holistic approach in caring for the patient. Assuming those who sustain a fall-related TBI conform to the traditional Stumbler paradigm fails to recognize a significant subset of patients we identified as Tumblers. Effective injury prevention lies in reducing psychosocial risk factors in many domains. Several strategies involving policy makers, mental health professionals, parents, teachers, and other adults can help address these risk factors early in development. For example, we have shown that pervasive violence, and substance and alcohol use can influence risk-taking in adulthood, and consequently the incidence of brain injuries. In turn, these brain injuries can be associated with adverse psychosocial and cognitive outcomes that exacerbate earlier life trajectories in susceptible individuals. Strategies and policies developed to provide safe early childhood trajectories through educational, environmental, economic, legislative, sporting, and engineering approaches could lay the foundation for healthier adult behaviors. Our results suggest that identifying children and families at high risk for adverse trajectories and providing targeted policies and strategies will likely show benefits well into adulthood, related not only to TBI occurrence, but also to other risky behaviors such as violence and substance abuse.

## Abbreviations Used

SD: standard deviation
TBI: traumatic brain injury

## References

[1] Leo, P., and McCrea, M. Epidemiology, in: Translational Research in Traumatic Brain Injury. D. Laskowitz, and G. Grant (eds). CRC Press/Taylor and Francis Group: Boca Raton, FL 26583170

[2] Das, A., Botticello, A.L., Wylie, G.R., and Radhakrishnan, K. (2012). Neurologic disability: a hidden epidemic for India. Neurology 79, 2146–2147 23170012 10.1212/WNL.0b013e3182752cdb PMC3511929

[3] Hyder, A.A., Wunderlich, C.A., Puvanachandra, P., Gururaj, G., and Kobusingye, O.C. (2007). The impact of traumatic brain injuries: a global perspective. NeuroRehabilitation 22, 341–353 18162698

[4] Public Health Agency of Canada. (2014). Mapping connections: an understanding of neurological conditions in Canada. http://www.phac-aspc.gc.ca/publicat/cd-mc/mc-ec/assests/pdf/mc-ec-eng.pdf (Last accessed 122, 2020).

[5] Elder, G.H. Jr. (1998). The life course as developmental theory. Child Dev. 69, 1–12 9499552

[6] Ilie, G., Adlaf, E.M., Mann, R.E., Ialomiteanu, A., Hamilton, H., Rehm, J., Asbridge, M., and Cusimano, M.D. (2018). Associations between self-reported lifetime history of traumatic brain injuries and current disability assessment in a population sample of Canadian adults. PLoS One 13, e0188908 29304117 10.1371/journal.pone.0188908 PMC5755742

[7] Brain Injury Association of America. (2006). Facts about traumatic brain injury. www.biausa.org/elements/aboutbi/factsheets (Last accessed 122, 2020)

[8] Cusimano, M.D., Saarela, O., Hart, K., Zhang, S., and McFaull, S.R. (2020). A population-based study of fall-related traumatic brain injury identified in older adults in hospital emergency departments. Neurosurg. Focus 49, E20 10.3171/2020.7.FOCUS20520 33002878

[9] Bogner, J.A., Corrigan, J.D., Mysiw, W.J., Clinchot, D., and Fugate, L. (2001). A comparison of substance abuse and violence in the prediction of long-term rehabilitation outcomes after traumatic brain injury. Arch. Phys. Med. Rehabil. 82, 571–577 11346830 10.1053/apmr.2001.22340

[10] Corrigan, J.D. (1995). Substance abuse as a mediating factor in outcome from traumatic brain injury. Arch. Phys. Med. Rehabil. 76, 302–309 7717829 10.1016/s0003-9993(95)80654-7

[11] Graham, D.P., and Cardon, A.L. (2008). An update on substance use and treatment following traumatic brain injury. Ann. N Y Acad. Sci. 1141, 148–162 18991956 10.1196/annals.1441.029

[12] Seigel, L.J. (2006). Criminology, 10th ed. Thomson Wadsworth: University of Massachusetts, Lowell

[13] Fattah, E.A. (1967). Vers une typologie criminoligique des victims. Revue Internationale de Police Criminelle 22, 162–169

[14] Meeuwisse, W.H. (1994). Assessing causation in sport injury: a multifactorial model. Clin. J. Sport Med. 4, 166–170

[15] Rothman, K.J. (1986). Modem Epidemiology. Little, Brown & Co.: Boston

[16] Last, J.M. (1998). A Dictionary of Epidemiology, 2nd ed. Oxford University Press: New York

[17] Elder, G.H. Jr., Johnson, M.K., and Crosnoe, R. (2003). The emergence and development of life course theory, in: Handbook of the Life Course. J.T. Mortimer, and M.J. Shanahan (eds). Springer: New York, pps. 3–19

[18] Ford, D.H., and Lemer, R.M. (1992). Developmental Systems Theory: An Integrative Approach. Sage Publications: London

[19] Hagestad, G.O. Parent and child: generations in the family, in: Review of Human Development. T.M. Field, A. Huston, A. Quay, L. Troll, and G.E. Finley (eds). Wiley: New York, pps. 485–499

[20] Featherman, D.L. (2014). The life-span perspective in social science research, in: Life-Span Development and Behavior. P.B. Baltes, and O.G. Brim (eds). Academic Press: New York, pps. 1–57

[21] Pennings, J.L., Bachulis, B.L., Simons, C.T., and Slazinski, T. (1993). Survival after severe brain injury in the aged. Arch. Surg. 128, 787–794 8317961 10.1001/archsurg.1993.01420190083011

[22] Strauss, A., and Corbin, J. (1990). Basics of Qualitative Research: Grounded Theory Procedures and Techniques. Sage Publications: Newbury Park, CA

[23] Kreutzer, J.S., Witol, A.D., and Marwitz, J.H. (1996). Alcohol and drug use among young persons with traumatic brain injury. J. Learn. Disabil. 29, 643–651 8942308 10.1177/002221949602900608

[24] Kolakowsky-Hayner, S.A., Gourley, E.V. III, Kreutzer, J.S., Marwitz, J.H., Cifu, D.X., and McKinley, W.O. (1999). Pre-injury substance abuse among persons with brain injury and persons with spinal cord injury. Brain Inj. 13, 571–581 10901686 10.1080/026990599121313

[25] Kreutzer, J.S., Wehman, P.H., Harris, J.A., Burns, C.T., and Young, H.F. (1991). Substance abuse and crime patterns among persons with traumatic brain injury referred for supported employment. Brain Inj. 5, 177–187 1873603 10.3109/02699059109008088

[26] Winqvist, S., Lehtilahti, M., Jokelainen, J., Luukinen, H., and Hillbom, M. (2007). Traumatic brain injuries in children and young adults: a birth cohort study from northern Finland. Neuroepidemiology 29, 136–142 17989501 10.1159/000110741

[27] Winqvist, S., Jokelainen, J., Luukinen, H., and Hillbom, M. (2006). Adolescents' drinking habits predict later occurrence of traumatic brain injury: 35-year follow-up of the northern Finland 1966 birth cohort. J. Adolesc. Health 39, 275.e1–7. 10.1016/j.jadohealth.2005.12.019 16892497

[28] Winqvist, S., Luukinen, H., Jokelainen, J., Lehtilahti, M., Nayha, S., and Hillbom, M. (2008). Recurrent traumatic brain injury is predicted by the index injury occurring under the influence of alcohol. Brain Inj. 22, 780–785 18787988 10.1080/02699050802339397

[29] Glaser, B.G., and Strauss, A.L. (1967). The Discovery of Grounded Theory: Strategies for Qualitative Research. Aldine: Chicago

[30] Charmaz, K.C. (2006). Constructing Grounded Theory: A Practical Guide through Qualitative Analysis. Sage Publications: Thousand Oaks, CA

[31] Wadhwa, P.D. (2015). Prenatal stress and life-span development, in: Encyclopedia of Mental Health. H.S. Friedman (ed). Academic Press: San Diego, CA, pps. 265–280

[32] Yehuda, R., Resnick, H., Kahana, B., and Giller, E.L. (1993). Long-lasting hormonal alterations to extreme stress in humans: normative or maladaptive? Psychosom. Med. 55, 287–297 8346336 10.1097/00006842-199305000-00006

[33] Natarajan, R., Northrop, N.A., and Yamamoto, B.K. (2015). Protracted effects of chronic stress on serotonin-dependent thermoregulation. Stress 18, 668–676 26414686 10.3109/10253890.2015.1087502 PMC4893822

[34] McEwen, B.S. (2003). Mood disorders and allostatic load. Biol. Psychiatry 54, 200–207 12893096 10.1016/s0006-3223(03)00177-x

[35] Duan, H., Yuan, Y., Zhang, L., Qin, S., Zhang, K., Buchanan, T.W., and Wu, J. (2013). Chronic stress exposure decreases the cortisol awakening response in healthy young men. Stress 16, 630–637 23992539 10.3109/10253890.2013.840579

[36] McEwen, B.S. (2001). Plasticity of the hippocampus: adaptation to chronic stress and allostatic load. Ann. N Y Acad. Sci. 933, 265–277 12000027 10.1111/j.1749-6632.2001.tb05830.x

[37] Stattin, H., and Magnusson, D. (1996). Antisocial development: a holistic approach. Dev. Psychopathol. 8, 617–645

[38] World Health Organization. (2002). World report on violence and health. https://www.who.int/violence_injury_prevention/violence/world_report/en/FullWRVH.pdf (Last accessed 122, 2020)

[39] Matherne, M.M., and Thomas, A. (2001). Family environment as a predictor of adolescent delinquency. Adolescence 36, 655–665 11928874

[40] Kadish, T.E., Glaser, B.A., Calhoun, G.B., and Risler, E.A. (1999). Counseling juvenile offenders: a program evaluation. J. Addict. Offend. Counsel 19, 88–94

[41] American Psychological Association. (1996). Violence and the family: report of the American Psychological Association presidential task force on violence and the family. https://eric.ed.gov/?id=ED399073 (Last accessed 122, 2020)

[42] English, D.J., Marshall, D.B., and Stewart, A.J. (2003). Effects of family violence on child behavior and health during early childhood. J. Fam. Violence 18, 43–57

[43] Wille, N., Bettge, S., Ravens-Sieberer, U., and the BELLA study group. (2008). Risk and protective factors for children's and adolescents' mental health: results of the BELLA study. Eur. Child Adolesc. Psychiatry 17, 133–147 10.1007/s00787-008-1015-y 19132313

[44] Eddy, J.M., Reid, J.B., and Fetrow, R.A. (2000). An elementary school-based prevention program targeting modifiable antecedents of youth delinquency and violence: Linking the Interests of Families and Teachers (LIFT). J. Emot. Behav. Disord. 8, 165–176

[45] Kwong, M.J., Bartholomew, K., Henderson, A.J., and Trinke, S.J. (2003). The intergenerational transmission of relationship violence. J. Fam. Psychol. 17, 288–301 14562454 10.1037/0893-3200.17.3.288

[46] Holden, G.W. (1998). Introduction: the development of research into another consequence of family violence, in: Children Exposed to Marital Violence: Theory, Research, and Applied Issues. G.W. Holden, R.A. Geffner, and E.N. Jouriles (eds). American Psychological Association: Washington, DC, pps. 1–18

[47] Fantuzzo, J.W., and Mohr, W.K. (1999). Prevalence and effects of child exposure to domestic violence. Future Child. 9, 21–32 10777998

[48] Rudo, Z.H., Powell, D.S., and Dunlap, G. (1998). The effects of violence in the home on children's emotional, behavioral, and social functioning: a review of the literature. J. Emot. Behav. Disord. 6, 94–113

[49] Pepler, D.J., Catallo, R., and Moore, T.E. (2000). Considering the children: research informing interventions for children exposed to domestic violence, in: Children Exposed to Domestic Violence: Current Issues in Research, Intervention, Prevention, and Policy Development. R. Geffner, P. Jaffe, and M. Sudermann (eds). The Haworth Maltreatment & Trauma Press: Binghamton, NY, pps. 37–57

